# Cost-minimization analysis of adjuvant chemotherapy regimens given to patients with colorectal cancer in Japan

**DOI:** 10.1186/s40780-016-0064-5

**Published:** 2016-11-09

**Authors:** Kosuke Takata, Ken-ichi Fujita, Yutaro Kubota, Hiroo Ishida, Wataru Ichikawa, Ken Shimada, Takashi Sekikawa, Iori Taki-Takemoto, Daisuke Kamei, Shinichi Iwai, Yasutsuna Sasaki

**Affiliations:** 1 Department of Healthcare and Regulatory Sciences, School of Pharmacy, Showa University, 1-5-8, Hatanodai, Shinagawa-ku, Tokyo, 142-8555 Japan; 2 Institute of Molecular Oncology, Showa University, 1-5-8, Hatanodai, Shinagawa-ku, Tokyo, 142-8555 Japan; 3 Division of Medical Oncology, Department of Internal Medicine, School of Medicine, Showa University, 1-5-8 Hatanodai, Shinagawa-ku, Tokyo, 142-8555 Japan; 4 Division of Medical Oncology, Department of Internal Medicine, Showa University Fujigaoka Hospital, 1-30 Fujigaoka, Aoba-ku, Yokohama, Kanagawa 227-8501 Japan; 5 Department of Internal Medicine, Showa University Koto Toyosu Hospital, 5-1-38 Toyosu, Koto-ku, Tokyo, 135-8577 Japan; 6 Department of Internal Medicine, Showa University Yokohama Northern Hospital, 35-1 Chigasakichuo, Tsuzuki-ku, Yokohama, Kanagawa 224-8503 Japan

**Keywords:** Cost-minimization analysis, Cost-effectiveness, Colorectal cancer, Adjuvant chemotherapy, Regimen selection

## Abstract

**Background:**

Consideration of medical costs as well as effectiveness and adverse events is rapidly been becoming an important factor in the selection of chemotherapy regimens. However, practical data on the costs of chemotherapy are scarce. We clinically estimated the medical costs of 6 adjuvant chemotherapy regimens for colorectal cancer on the basis of clinical and cost-related data and compared their cost-effectiveness by cost-minimization analyses.

**Methods:**

All patients who received adjuvant chemotherapy for colorectal cancer between April 2012 and May 2015 at four hospitals affiliated with Showa University were studied retrospectively. Clinical and cost data related to adjuvant chemotherapy were collected from medical records and medical fee receipt data, respectively. Six adjuvant chemotherapy regimens were studied: capecitabine and oxaliplatin (CapeOX); 5-fluorouracil (5-FU), ℓ-leucovorin (LV), and oxaliplatin (modified FOLFOX6 [mFOLFOX6]); 5-FU and LV (5-FU/LV); tegafur and uracil (UFT), and LV (UFT/LV); capecitabine; and tegafur, gimeracil and oteracil (S-1). The regimens were divided into 2 groups according to whether or not they contained oxaliplatin because of the difference in effectiveness. Cost-minimization analyses, where relative costs of regimens showing equivalent effectiveness were simply compared, were performed to evaluate the cost-effectiveness of the regimens in each group.

**Results:**

A total of 154 patients with colorectal cancer received adjuvant chemotherapy during the study period. Fifty-seven patients were treated with CapeOX, 10 with mFOLFOX6, 38 with UFT/LV, 20 with capecitabine, and 29 with S-1. No patient received 5-FU/LV. The total costs of oxaliplatin-containing regimens were significantly higher than those of oxaliplatin non-containing regimens. The high cost of oxaliplatin, but not the costs of drugs or various tests for the treatment of adverse events, was the primary reason for the higher costs of the oxaliplatin-containing regimens. The cost-effectiveness of the oxaliplatin-containing regimens CapeOX and mFOLFOX6 were comparable. Among the oxaliplatin non-containing regimens, the cost-effectiveness of S-1 and capecitabine was superior to that of UFT/LV.

**Conclusion:**

Thus, we provided the cost-effectiveness data of 5 adjuvant chemotherapy regimens for colorectal cancer based on practical clinical and cost data from Japanese patients. The results can be included as a factor in regimen selection because these results would represent the real world.

**Trial registration:**

This study is a retrospective observational study and does not include any health care interventions. Therefore, we did not register the protocol of this study.

## Background

Cancer therapy has rapidly evolved over the past two decades, contributing to improvements in the survival and quality of life of cancer patients. However, the costs of the cancer therapy have also rapidly increased in parallel to progress in cancer therapy [1]. A previous study reported that 30.6 % or more of patients with cancer are complaining about the rising costs of cancer therapy [2]. Another study found that the frequency of bankruptcy was 2.65-fold higher among patients with cancer than those without the disease [3]. Many highly effective anticancer drugs have recently been developed and are now used in clinical practice. However, the costs of these drugs are generally high. For example, the cost of one intravenous dose of the cytotoxic anticancer drug oxaliplatin is higher than 80,000 yen (800 US dollars, assuming that 100 yen is equivalent to 1 dollar) when the drug is given to a Japanese patient with an average body surface area (BSA) of 1.69 m^2^ [4]. As for molecularly targeted drugs, the cost of one dose of bevacizumab or cetuximab is higher than 100,000 yen (1000 dollars). In the case of the immune checkpoint inhibitor nivolumab, which was very recently launched, the cost of a single intravenous dose of the drug exceeds 1,000,000 yen (10,000 dollars). Given the remarkable increase in the costs of anticancer drug therapies, oncologists can no longer ignore or blindly accept that costs have no place in medical decision making [5]. Therefore, it has been widely recommended that costs related to cancer chemotherapy should be considered in addition to effectiveness and adverse events in the selection of treatment regimens [5, 6]. However, cost data on cancer medications in Japan are extremely limited; patients and oncologists generally choose treatment regimens on the basis of only effectiveness and adverse events, without considering costs.

For patients who have pathological stage II colorectal cancer with a high risk of recurrence or patients who have stage III disease, adjuvant chemotherapy is recommended after potentially curative resection [7]. Six adjuvant chemotherapy regimens are used to treat colorectal cancer in Japan: 1) CapeOX, consisting of capecitabine and oxaliplatin [8]; 2) FOLFOX4, comprising 5-fluorouracil (5-FU), ℓ-leucovorin (LV), and oxaliplatin [9], which is usually replaced by modified FOLFOX6 (mFOLFOX6), comprising the same agents as FOLFOX4, in Japan, because mFLOFOX6 is simpler to handle than FOLFOX4, while the effectiveness and safety of these regimens are nearly equivalent [10]; 3) 5-FU/LV, consisting of 5-FU plus LV [11]; 4) UFT/LV, comprising UFT (a fixed combination of tegafur and uracil) and oral LV [12]; 5) capecitabine [13]; and 6) S-1 (tegafur, gimeracil, and oteracil) [14].

Several economic studies have examined the cost-effectiveness of adjuvant chemotherapy for colorectal cancer in Japan [15–17]. The clinical data used in these studies were derived from international phase 3 trials, but not based on clinical practice. The cost of a drug or a test was calculated by multiplying the pre-determined numbers of drug doses or tests by their respective unit prices. These methods have the advantage that cost calculation is straightforward and simple. However, the costs related to adjuvant chemotherapy thus obtained might differ from those obtained by using patient data in the real world, because patients’ backgrounds are different between international phase 3 trials and clinical practice. In clinical practice, subpopulations of patients with advanced age, comorbidities, organ dysfunctions, or lower performance status who generally cannot participate in international phase 3 trials are given adjuvant chemotherapy. Given that patients who receive adjuvant chemotherapy in clinical practice might receive a lower dose intensity and suffer more severe adverse events than patients enrolled in international phase 3 trials, considerable differences in the medical costs from the phase 3-based approach are plausible. When selecting regimens for patients in clinical practice, the use of the medical costs reflecting the actual situation is desirable.

Based on these backgrounds, we calculated the total costs of 6 regimens of adjuvant chemotherapy for colorectal cancer by using data from Japanese patients treated in clinical practice. Based on the costs thus obtained, we compared the cost-effectiveness of these regimens.

## Methods

This was a retrospective study of all patients who received adjuvant chemotherapy for colorectal cancer in Showa University Hospital, Showa University Fujigaoka Hospital, Showa University Koto Toyosu Hospital, or Showa University Northern Yokohama Hospital between April 2012 and May 2015. The present study was approved by the Institutional Review Board of Showa University (approved number; Showa University Hospital, 1824; Showa University Fujigaoka Hospital, 2015023; Showa University Koto Toyosu Hospital, 15T7006; Showa University Northern Yokohama Hospital, 1505-07).

### Selection of patients

All patients who received either CapeOX, mFOLFOX6, 5-FU/LV, UFT/LV, capecitabine, or S-1 at the aforementioned hospitals and completed all scheduled cycles were studied. Patients were required to undergo potentially curative resection for colorectal cancer before receiving adjuvant chemotherapy.

### Chemotherapeutic regimens

CapeOX consisted of a 2-h intravenous infusion of oxaliplatin (130 mg/m^2^) on day 1 and oral capecitabine (1000 mg/m^2^) twice daily on days 1 to 14, repeated every 3 weeks for 8 cycles [8]. mFOLFOX6 consisted of LV (200 mg/m^2^) given as a 2-h infusion and oxaliplatin (85 mg/m^2^) given as a 2-h infusion, followed by a bolus infusion of 5-FU (400 mg/m^2^) and a 46-h continuous infusion of 5-FU (2400 mg/m^2^). This regimen was repeated every 2 weeks for 12 cycles [10]. Brand-name oxaliplatin was used in CapeOX and mFOLFOX6. 5-FU/LV comprised a 2-h infusion of LV (250 mg/m^2^) and a bolus infusion of 5-FU (500 mg/m^2^) given 1 h after starting the LV infusion, repeated weekly for 6 weeks followed by a 2-week rest [11]. This regimen was given for 3 cycles. UFT/LV consisted of oral UFT (300 mg/m^2^) and LV (75 mg/patient) given 3 times daily on days 1 to 28 followed by a 7-day rest, repeated for 5 cycles [12]. Capecitabine was given orally in a dose of 1250 mg/m^2^ twice daily on days 1 to 14, followed by a 7-day rest, repeated for 8 cycles [13]. S-1 was administered orally twice daily for 28 consecutive days, followed by a 2-week rest. S-1 was given in a fixed dose based on the patient’s BSA according to the dose recommendations of the manufacturer’s package insert in Japan. The dose was 80 mg/day for patients with a BSA of less than 1.25 m^2^, 100 mg/day for those with a BSA of 1.25 to 1.5 m^2^, and 120 mg/day for those with a BSA of more than 1.5 m^2^. This regimen was given for 4 cycles [14].

### Data collection

Patient background data, such as age and disease stage, as well as data during adjuvant chemotherapy, including laboratory tests, prescribed drugs, and adverse events, were collected from the patients’ medical records.

Cost data related to adjuvant chemotherapy were extracted from medical fee receipt data. Costs for outpatient visits, laboratory tests, imaging tests for tumor diagnosis, and prescription fees for administered drugs were collected. The cost of each administered drug was calculated by multiplying the drug dose prescribed by its unit price according to the Japanese National Health Insurance fee-for-service system in 2014. The summation of these costs was defined as total cost. Since all hospitals in Showa University have adopted the diagnosis procedure combination (DPC) system [18], hospitalization costs were constant regardless of the number of drugs administered and laboratory tests performed. When the total hospitalization costs calculated by the DPC included the cost of drugs related to adjuvant chemotherapy, the drug costs were calculated by the method described above (the drug dose prescribed x its unit price), and the hospitalization cost was calculated by subtracting the cost of chemotherapy-related drugs from the hospitalization cost according to the DPC. This analysis was performed from the perspective of the health care payer. We described the unit of all costs by Japanese yen and US dollars, assuming that 1 US dollar was equivalent to 100 Japanese yen.

### Cost-minimization analyses

**Fig. 1:**
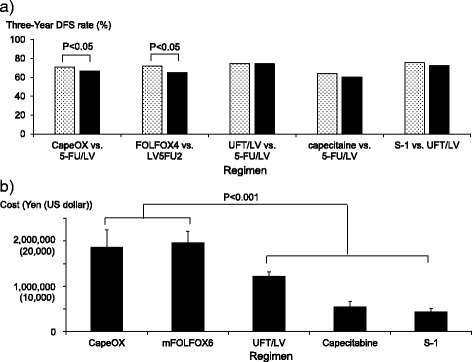
Comparisons of **a**) effectiveness and **b**) total costs among adjuvant chemotherapy regimens for colorectal cancer. **a** Three-year DFS rates of CapeOX and FOLFOX4 were superior to that of 5-FU containing regimens [8, 9], whereas those of UFT/LV and capecitabine showed non-inferiority to 5-FU containing regimens [12, 13] (see Methods session). S-1 was non-inferior to UFT/LV [14] (see Methods session). **b** The total costs included anticancer drug costs, hospitalization costs, laboratory and imaging test costs, prescription fees for administered drugs, supportive care drug costs, and other costs. The total costs of oxaliplatin-containing regimens were significantly higher than those of oxaliplatin non-containing regimens (*P* < 0.001). Mean ± standard deviation, *n* = 57 for CapeOX, *n* = 10 for mFOLFOX6, *n* = 38 for UFT/LV, *n* = 20 for capecitabine, *n* = 29 for S-1

Cost-minimization analysis is one of methods to evaluate cost-effectiveness of therapeutic options [19], in which relative costs of therapeutic options showing equivalent outcomes of interventions are simply compared. We performed cost-minimization analyses for the oxaliplatin-containing regimens (CapeOX and mFOLFOX6) and the oxaliplatin non-containing regimens (5-FU/LV, UFT/LV, capecitabine, and S-1) because of the following reasons:

1) Because there was no direct comparison between CapeOX and mFOLFOX6, we compared the effectiveness of these regimens based on the following considerations. As demonstrated by 2 international phase 3 trials, 16968 [8] and MOSAIC [9], the effectiveness of CapeOX and FOLFOX4 was significantly superior to that of 5-FU/LV and LV5FU2, respectively (Table 1 and Fig. 1a)). Because the effectiveness of LV5FU2 and 5-FU/LV [20, 21] and that of FOLFOX4 and mFOLFOX6 were comparable [10] (Table 1), the 3-year disease-free survival (DFS) rates of both CapeOX and mFOLFOX6 were comparable and approximately 5 % higher than that of 5-FU/LV. 2) Two international phase 3 trials, NSABP C-06 [12] and X-ACT [13] (Table 1), showed that UFT/LV and capecitabine were noninferior to 5-FU/LV in terms of 5-year overall survival (OS). In addition, the ACTS-CC international phase 3 trial demonstrated that S-1 was noninferior to UFT/LV with respect to the 3-year DFS rate [14] (Table 1 and Fig. 1a)). On the basis of these results, we assumed that the effectiveness of these 3 regimens was comparable and nearly equivalent to the effectiveness of 5-FU/LV.

**Table 1:** Phase 3 trials of adjuvant chemotherapy for colorectal cancer

Trials	Race	Regimens	Primary endpoint	Result of the trials	Conclusion of the trials	Reference
16968	Whites	5-FU/LV vs. CapeOX	3-Year DFS rate	66.5 vs. 70.9 %	Superiority of CapeOX to 5-FU/LV	[8]
MOSAIC	Whites	LV5FU2 vs. FOLFOX4	3-Year DFS rate	65.3 vs. 72.2 %	Superiority of FOLFOX4 to LV5FU2	[9]
INT 0089	Whites	5-FU/LV (RPMI) vs. 5-FU/LV (Mayo)	5-Year OS rate	66.0 vs. 66.0 %	Non-inferiority of 5-FU/LV (RPMI) to 5-FU/LV (Mayo)	[20]
GERCOR C96.1	Whites	5-FU/LV (Mayo) vs. LV5FU2	6-Year DFS rate	65.0 vs. 66.0 %	Non-inferiority of 5-FU/LV (Mayo) to LV5FU2	[21]
	Japanese	FOLFOX4 vs. mFOLFOX6	Response rate	53.7 vs. 46.6 %	Non-inferiority of mFOLFOX6 to FOLFOX4	[10]^a^
NSABP C-06	Whites	5-FU/LV vs. UFT/LV	5-Year OS rate	71.5 vs. 69.6 %	Non-inferiority of UFT/LV to 5-FU/LV	[12]
X-ACT	Whites	5-FU/LV vs. capecitabine	3-Year DFS rate	60.6 vs. 64.2 %	Non-inferiority of capecitabine to 5-FU/LV	[13]
ACTS-CC	Japanese	UFT/LV vs. S-1	3-Year DFS rate	72.5 vs. 75.5 %	Non-inferiority of S-1 to UFT/LV	[14]

*RPMI* Roswell Park Memorial Institute regimen

^a^Phase 2 trial

### Statistical analyses

Differences in quantitative variables, including cost data, were tested using the nonparametric Wilcoxon rank-sum test. Differences in qualitative variables were tested using the *χ* ^2^ test. Two-tailed *P* values of less than 0.05 were considered to indicate statistical significance. All analyses were carried out with the use of JMP version 12.0 software (SAS Institute, Cary, NC).

## Results

### Patient characteristics

From April 2012 through May 2015, a total of 154 patients with colorectal cancer received adjuvant chemotherapy in hospitals affiliated with Showa University. Fifty-seven patients were treated with CapeOX, 10 with mFOLFOX6, 38 with UFT/LV, 20 with capecitabine, and 29 with S-1 (Table 2). No patient was given 5-FU/LV during the study period. The distributions of gender, age, site of cancer, and performance status were similar among the 5 regimens. The stage of cancer significantly differed among these regimens (*P* < 0.001). Ratios of patients with stage III in CapeOX and mFOLFOX6 were higher than those in UFT/LV, capecitabine, and S-1.

**Table 2:** Patient characteristics

	CapeOX	mFOLFOX6	UFT/LV	Capecitabine	S-1	*P*
Gender†						
Male/female	32/25	5/5	20/18	10/10	18/11	0.909^a^
Age‡	65.0 (79-40)	55.5 (73-41)	67.0 (79-40)	60.0 (78-40)	63.0 (80-42)	0.309^b^
Tumor type						
Colon cancer/rectal cancer†	35/22	9/1	27/11	15/5	17/12	0.372^a^
Stage†						
I / II / III	0/3/54	0/0/10	0/11/27	0/2/18	4/11/14	<0.001^a^
Performance status†						
0/1	57/0	10/0	35/3	18/2	29/0	0.0680^a^

†Number; ‡Median (range)

^a^χ^2^ test; ^b^Analysis of variance

### Cost analyses

Total costs calculated for each regimen are shown in Fig. 1b). The costs of oxaliplatin-containing regimens were approximately 1,860,000 yen (18,600 dollars) for CapeOX and 1,970,000 yen (19,700 dollars) for mFOLFOX6. The total costs of oxaliplatin-containing regimens were significantly higher than those of oxaliplatin non-containing regimens (*P* < 0.001) (CapeOX vs. UFT/LV, *P* < 0.001; CapeOX vs. capecitabine, *P* < 0.001; CapeOX vs. S-1, *P* < 0.001; mFOLFOX6 vs. UFT/LV, *P* < 0.001; mFOLFOX6 vs. capecitabine, *P* < 0.001; mFOLFOX6 vs. S-1, *P* < 0.001) (Fig. 1b). The total costs of CapeOX and mFOLFOX6 did not differ significantly (*P* = 0.374).

Among the oxaliplatin non-containing regimens, the total cost of UFT/LV was significantly higher than that of capecitabine (*P* < 0.001). The cost of capecitabine was significantly higher than that of S-1 (*P* = 0.003).

### Factors causing the higher costs of oxaliplatin-containing regimens

**Fig. 2:**
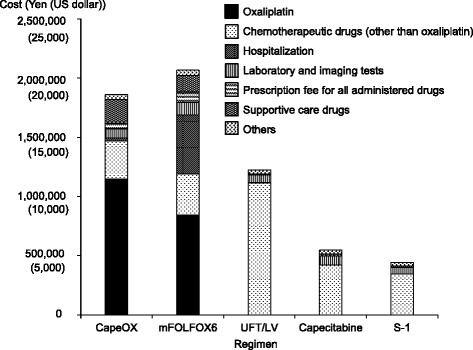
Breakdown of the total costs for each regimen. Supportive care drugs included drugs used as premedication to prevent nausea and vomiting, drugs used to treat adverse events, and infusion solutions (see Fig. 3)

**Fig. 3:**
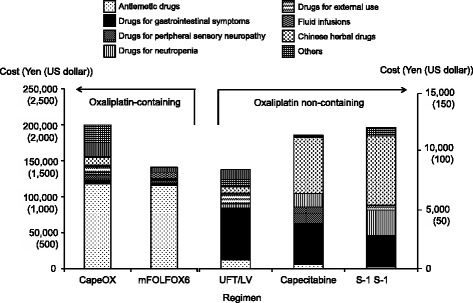
Breakdown of the costs for drugs prescribed for supportive care in each regimen. Representative therapeutic drugs included in Others for CapeOX were ELENTAL®, KRESTIN®, levofloxacin, loxoprofen, and Posterisan® forte

To address the causes of the higher total costs of oxaliplatin-containing regimens, the breakdown of the costs for each regimen was calculated (Fig. 2). The cost of oxaliplatin in CapeOX was about 1,150,000 yen (11,500 dollars), which was equivalent to approximately 60 % of the total cost. In the case of mFOLFOX6, the cost of oxaliplatin was about 900,000 yen (9000 dollars), which was equivalent to approximately 40 % of the total cost. The total cost of mFOLFOX6 also included hospitalization costs (400,000 yen [4000 dollars]), such as the fee required to prepare a central venous port for administration of 5-FU, LV, and oxaliplatin. Thus, the hospitalization costs required for mFOLFOX6 increased the total cost of this regimen to a level comparable to the cost of CapeOX. The costs of drugs for supportive care required to administer CapeOX and mFOLFOX6 were approximately equivalent to 10 % of the total costs. The breakdown of the costs of supportive care drugs is shown in Fig. 3. The costs of the drugs prescribed to treat peripheral sensory neuropathy, which is frequently associated with oxaliplatin-related chemotherapy, were approximately 7500 yen (75 dollars) for CapeOX and 4300 yen (43 dollars) for mFOLFOX6, which comprised only 0.4 and 0.2 % of the total costs of CapeOX and mFOLFOX6, respectively. We considered the possibility that a lower frequency of peripheral sensory neuropathy in the present study than in previous studies led to the lower cost of prescriptions for this adverse event. The frequency of peripheral sensory neuropathy of CapeOX in the present study was lower than the results of previous study (Table 3). However, in the case of mFOLFOX6, the frequency and grade of peripheral sensory neuropathy in the present study were not necessarily lower than those of previous studies (Table 3). On the other hand, the costs of antiemetics were approximately 118,000 yen (1180 dollars) for CapeOX and 116,000 yen (1160 dollars) for mFOLFOX6, accounting for about 6 % of the total costs. Antiemetics such as aprepitant, azasetron, domperidone, granisetron, metoclopramide, ondansetron, palonosetron, prochlorperazine and ramosetron were prescribed in CapeOX and mFOLFOX6 regimens. The parentages of patients who used palonosetron and aprepitant were 100 and 26 % in CapeOX, and 60 and 40 % in mFOLFOX6, respectively.

**Table 3:** Comparison of the frequency of peripheral sensory neuropathy between present study and phase 3 trials

Regimen	Grade	Present study	Phase 3 trials
CapeOX	All Grade	54.4 %	78.0 %^a^
	≥ Grade 3	1.80 %	11.0 %^a^
mFOLFOX6	All Grade	90.0 %	92.0 %^b^
	≥ Grade 3	40.0 %	12.5 %^b^

Grade of neuropathy was evaluated according to the Common Terminology Criteria for Adverse Events version 3.0.

^a^Data from reference [8]; ^b^Result of FOLFOX4 [9]. Effectiveness and safety of mFOLFOX6 were comparable to those of FOLFOX4 [10].

### Cost-minimization analyses

Because the effectiveness (Methods session and Fig. 1a)) and the total costs (Fig. 1b)) of CapeOX and mFOLFOX6 were comparable, the cost-effectiveness of these regimens was judged to be similar (Table 4). As described in the Methods session and Fig. 1a), the effectiveness of the oxaliplatin non-containing regimens was comparable. Therefore, on the basis of the total costs of these regimens (Fig. 1b)), the cost-effectiveness of S-1 was superior to that of UFT/LV, and the cost-effectiveness of capecitabine was superior to that of UFT/LV, which were caused by the high cost of LV.

**Table 4:** Cost-minimization analyses

Regimen	Comparison of cost	Comparison of effectiveness	Cost-minimization analyses
CapeOX vs. mFOLFOX6	Comparable	Comparable	Comparable
UFT/LV vs. S-1	Higher in UFT/LV than S-1	Comparable	S-1 superior to UFT/LV
UFT/LV vs. capecitabine	Higher in UFT/LV than capecitabine	Comparable	Capecitabine superior to UFT/LV

## Discussion

The present study compared the cost effectiveness of 5 regimens of adjuvant chemotherapy given to patients with colorectal cancer. The total costs were calculated with the use of clinical and cost data obtained from Japanese patients who received each regimen of adjuvant chemotherapy in clinical practice. This is in contrast to most previous studies assessing the costs of adjuvant chemotherapy for colorectal cancer in Japan, which based the costs of treatment on clinical data obtained from large phase 3 clinical trials [15–17].

To date, three studies of cost-effectiveness employing clinical data from phase 3 clinical trials have been performed: Hisashige et al. [15] analyzed the cost-effectiveness of UFT by comparing clinical and cost data between patients who received or did not receive UFT in the NSAS CC trial [22]. In other Japanese studies, the cost-effectiveness of 5-FU/LV and capecitabine [16] was evaluated with the use of clinical data from X-ACT trial [13], and that of 5-FU/LV and FOLFOX4 [17] was evaluated with the use of data from the MOSAIC trial [9]. We compared the costs required for the following 3 categories between the present study and previous studies based on large international phase 3 trials: 1) anticancer drugs, 2) drugs used for supportive care, and 3) laboratory tests. 1) The previously estimated cost of 1 year of treatment with UFT (about 393,700 yen [3937 dollars]) [15] was generally similar to the cost calculated by us (i.e., about 360,200 yen [3602 dollars], equivalent to twice the cost of 6 months’ treatment with UFT in our study). However, the cost of capecitabine calculated in a previous study (540,000 yen [5400 dollars]) [16] was higher than that estimated by us (about 420,500 yen [4205 dollars]). The reason for the higher cost of capecitabine in the previous study is considered to be the difference in relative dose intensity (RDI) of capecitabine between the two studies. The previous study used a theoretical RDI of 100.0 %, whereas our study used the clinically observed RDI of 75.4 %. The cost of capecitabine estimated by Shiroiwa et al. [16] would have been about 407,200 yen (4072 dollars) if an RDI of 75.4 % had been adopted, which is nearly comparable to our estimated cost. 2) The costs of agents prescribed for supportive care in previous studies of UFT and capecitabine [15, 16] were about 300 yen (3 dollars) and 7000 yen (70 dollars), respectively, while those in the present study were about 8400 yen (84 dollars) for UFT/LV and about 17,500 yen (175 dollars) for capecitabine, demonstrating clearly higher costs for supportive care in our study. The primary reason first considered for the higher supportive care costs in our study was a higher incidence of adverse events in the present study than in previous studies. However, the incidence of bilirubin increase in the NSAS CC trial was 60.0 % [22], as compared with 10.5 % in the present study. The incidence of hand-foot syndrome associated with capecitabine regimens was 60.0 % in the X-ACT trial [13] and 30.0 % in our study. Thus, the incidences of adverse events were not necessarily higher in our study as compared with previous phase 3 trials. As shown in Fig. 3, patients given UFT/LV were mainly prescribed drugs to manage gastrointestinal symptoms, such as proton pump inhibitors and histamine-2 blockers. In patients who received capecitabine, Chinese herbal drugs such as Juzentaihoto and Hochuekkito were predominantly prescribed. The costs of these drugs might have contributed to the higher costs for supportive care drugs in our study. 3) The estimated cost of laboratory tests for UFT regimens in a previous study (about 180,100 yen [1801 dollars]) [15] was approximately 3 times higher than that calculated in our practical study (about 65,500 yen [655 dollars]). On the other hand, the laboratory test costs in patients who received FOLFOX4 regimens in a previously reported study (76,800 yen [768 dollars]) [17] was lower than that in our present study (about 106,500 yen [1065 dollars]). These findings indicate that the costs of 1) anticancer drugs, 2) drugs prescribed for supportive care, and 3) laboratory tests calculated on the basis of clinical data from phase 3 trials differ from those calculated on the basis of data from actual clinical practice. Because the costs calculated from patient data in clinical practice would precisely represent the actual situation, cost-effectiveness data thus obtained can be used for regimen selection.

In Japan, a system of the public health insurance for the entire nation has been adopted. Patients have to pay for medical costs according to their age and income. The cost borne by the patient ranges from 10.0 to 30.0 % of total medical costs. In addition, the patient’s financial burden is maintained below specified limits under the high-cost medical care benefit system. The specified limits are determined by the patient’s income. If this system is applied, the costs for adjuvant chemotherapy that would be actually paid by the patient could be lower. Data from Showa University Hospital indicate when the public health insurance was applied to a patient, the cost of oxaliplatin-containing regimens was approximately 550,000 yen (5500 dollars), and that of UFT/LV was 263,000 yen (2630 dollars). The difference was 287,000 yen (2870 dollars). However, when the specified limits were applied, the cost of oxaliplatin-containing regimens was approximately 448,000 yen (4480 dollars), and that of UFT/LV was approximately 262,000 yen (2620 dollars), leading to a difference of 186,000 yen (1860 dollars). Thus, the specified limits might lower the medical costs of oxaliplatin-containing regimens to a greater extent than the costs of UFT/LV, although the specified limits system is not necessarily applicable to all patients because application of this system depends on the income of each patient. It is plausible that patients who derive an economic benefit tend to select oxaliplatin-containing regimens over other regimens. The medical costs are supplemented with taxes from Japanese citizens. To maintain the patient’s financial burden below specified limits, Japanese citizens have to pay higher taxes. This is an important issue to be discussed by health care payer.

An analysis of patient characteristics showed the stage of cancer significantly differed among the regimens (Table 2). However, the total costs of the CapeOX, UFT/LV, and S-1 regimens did not differ significantly between stage II and stage III. (*P* = 0.668, *P* = 0.711, and *P* = 0.743, respectively). Therefore, there might be no relation between the stage of cancer and total costs.

Our study had several limitations. 1) Direct comparisons of effectiveness are not available for some of the regimens. For example, no phase 3 trials have compared effectiveness between CapeOX and mFOLFOX6 or between UFT/LV and capecitabine. We therefore compared the effectiveness of CapeOX and mFOLFOX6 by the indirect comparisons of independent phase 3 trials (see Methods session). 2) The phase 3 trials that we referred to when comparing the effectiveness of the regimens were not necessarily performed in Japan. Theoretically, the effectiveness of the regimens should have been compared on the basis of data from phase 3 trials performed in Japan; however, we used data from clinical trials performed in whites because suitable Japanese trials were unavailable. It is well known that the survival advantage of a specific regimen in Japanese trials is generally better than that in clinical trials performed in other countries. For example, trials conducted in only Japanese patients tend to have better 3-year DFS rates and 5-year OS rates than those performed in whites [23]. One of the reasons is thought to be the better operation quality in Japan. For example, the extent of lymph-node resection during cancer surgery is greater in Japan than in other countries. 3) Some of the phase 3 trials that we referred to when comparing the effectiveness of the regimens included patients with stage III, but others included those with stage II and stage III. The effectiveness of these phase 3 trials might be affected by the difference in stage of patients enrolled. Taken together, our comparisons of the effectiveness of different regimens might have been biased by such factors.

## Conclusions

Costs of oxaliplatin-containing regimens were significantly higher than those of oxaliplatin non-containing regimens, but the cost-effectiveness of the oxaliplatin-containing regimens CapeOX and mFOLFOX6 were judged to be comparable. Among the oxaliplatin non-containing regimens, the cost-effectiveness of S-1 and capecitabine were superior to that of UFT/LV. Costs based on clinical data from phase 3 trials were shown to differ from costs based on data from actual clinical practice. Because costs based on patient data in clinical practice would more precisely represent the actual situation, the resulting cost-effectiveness data can be used for regimen selection.

## Abbreviations

5-FU: 5-Fluorouracil
5-FU/LV: 5-FU and LV
BSA: Body surface area
CapeOX: Capecitabine and oxaliplatin
DFS: Disease-free survival
DPC: Diagnosis procedure combination
FOLFOX4 or FOLFOX6: 5-FU, LV, and oxaliplatin
LV: ℓ-Leucovorin
mFOLFOX6: Modified FOLFOX6
OS: Overall survival
RDI: Relative dose intensity
S-1: Tegafur, gimeracil, and oteracil
UFT: Tegafur and uracil
UFT/LV: UFT, and oral LV
